# The Impact of COVID-19-Related Stress on Diet and Eating Behaviors in US College Students: A Cross-Sectional Study

**DOI:** 10.21203/rs.3.rs-6196663/v1

**Published:** 2025-03-14

**Authors:** Olufisayo Atanda-Ogunleye, Shuxian Hua, Bianca Borsarini, Sarah Ann Duck, Elena Jansen, Susan Carnell

**Affiliations:** Johns Hopkins University School of Medicine; Johns Hopkins University School of Medicine; Johns Hopkins University School of Medicine; Johns Hopkins University School of Medicine; Johns Hopkins University School of Medicine; Johns Hopkins University School of Medicine

**Keywords:** COVID-19 pandemic, stress, eating behaviors, dietary intake, nutrition, academic performance, young adults

## Abstract

**Background:**

The COVID-19 pandemic exposed the US population, including college students, to stress posing challenges to psychological and behavioral health. Previous studies have demonstrated that stress can promote unhealthy eating behaviors among college students. This study aimed to examine the relationships of pandemic-related stress with changes in diet and eating behaviors experienced by college students during the Fall 2020 semester.

**Methods:**

758 college students in the Mid-Atlantic region of the US completed an online survey in November 2020. The survey assessed multiple dimensions of pandemic-related stress, diet, and eating behaviors, as well as measures of psychological health and social support.

**Results:**

Pandemic-related stress, particularly academic stress, was correlated with less healthy dietary profiles and potentially maladaptive eating behaviors, including emotional eating and late-night eating. Associations between stress and dietary intake were stronger in females than males, whereas males showed stronger associations between stress and food responsiveness. Pandemic-related stress was associated with perceived changes in diet quality, frequency of eating, and amount of food consumed compared to since before the semester started.

**Conclusion:**

Academic stress during the pandemic had a negative impact on diet and eating behaviors among college students. Our results argue for interventions targeting academic stress in everyday contexts as well as potential future public health crises, to prevent negative impacts on students’ eating profiles that may in turn negatively impact health.

## 1. Introduction

The COVID-19 pandemic caused a global decline in psychological health, resulting in what some have described as a psychiatric epidemic (Gloster et al., 2020; Hossain et al., 2020). Evidence suggests that populations experienced increasing levels of stress, depression, and anxiety stemming from pandemic-related psychosocial stressors such as diminished social support and anxiety regarding COVID-19 exposure (Gloster et al., 2020; Hossain et al., 2020). College students are known to experience poor psychological health outcomes, and the pandemic introduced additional stressors impacting both their academic and personal lives (Wang et al., 2023). During periods of heightened stress, college students are susceptible to changes in dietary intake and eating habits (Errisuriz et al., 2016; Pelletier et al., 2016). For instance, increased stress among college students has been associated with changes in eating patterns that may negatively impact health, including increased rates of meal skipping and late-night eating (Pelletier et al., 2016), as well as greater consumption of unhealthy snacks and fast food (Errisuriz et al., 2016). Such dietary and behavioral changes during young adulthood can have long-term consequences, including elevated risks of type 2 diabetes and poorer mental health, as suggested by other studies of COVID-19 impacts (Hussein & Soliman, 2023; Rogers et al., 2021).

Despite the uniquely stressful experiences college students underwent during the pandemic, the impact of COVID-19-related stress on their diet and eating behaviors has not been comprehensively investigated. A few studies found that social isolation and online learning during the pandemic posed challenges (e.g., changes to learning environments, job searches, and the ability to afford treatments) and resulted in increased stress with concomitant effects on diet and eating behaviors among college students (Amatori et al., 2020; Huber et al., 2021; LaCaille et al., 2021). However, findings have been heterogeneous, with some reports of increased consumption of bread and sweets, decreased intake of legumes and low-fat meat, and larger portion sizes, but other reports of healthier dietary choices such as eating more fiber-rich foods, like fruit and vegetables (Amatori et al., 2020; Dhammawati et al., 2023; Huber et al., 2021; LaCaille et al., 2021). These conflicting results highlight the complexity of stress-related dietary responses and the potential role of individual differences in eating behaviors during the pandemic. Although changes in diet and eating behaviors among youth during the pandemic have been extensively studied across different cultural contexts (Amatori et al., 2020; Galali, 2021; Huber et al., 2021; Imaz-Aramburu et al., 2021; Jia et al., 2021; Kołota & Głąbska, 2021; LaCaille et al., 2021), few studies have examined how these changes relate to underlying psychological factors, such as stress (Elsalem et al., 2020). Furthermore, much of the existing research has focused on documenting behavioral shifts without identifying the specific stressors responsible for these changes among young populations (Imaz-Aramburu et al., 2021; Jehi et al., 2023; Jia et al., 2021; Kołota & Głąbska, 2021; Pung et al., 2021). For example, compared to before the pandemic, healthier eating behaviors and more consistent meal patterns were reported in some studies (Duong et al., 2020; Rafraf et al., 2023), while others have reported an increased intake of high-energy foods and less healthy foods, along with higher rates of binge eating, emotional eating, meal skipping, unhealthy snacking, and food insecurity (Coakley et al., 2021; Elsalem et al., 2020; Jehi et al., 2023; Pung et al., 2021). These inconsistencies underscore the need for further research to examine how different aspects of COVID-19-related stress (e.g., academic pressures, financial hardships, risk of exposure) may have influenced diet and eating behaviors among college students.

The current study aimed to address some of the research gaps outlined above by comprehensively investigating the relationships of COVID-19-related stress with dietary intake and eating behaviors via a survey conducted in a large, ethnically diverse sample of US undergraduates during the Fall 2020 semester. Our overarching hypothesis was that increased pandemic-related stress would be associated with higher consumption of less healthy foods (e.g., sweets, savory snacks, fast food, sugar-sweetened beverages) and potentially adverse eating behaviors (e.g., emotional eating, emotional overeating, and late-night eating). Based on previous evidence suggesting stronger relationships between stress and both eating behaviors of food eaten among women (Degroote et al., 2024; Zellner et al., 2006), and higher rates of binge eating in women (Rosenbaum & White, 2015), we additionally explored sex differences in associations of stress with diet and eating behaviors.

## 2. Methods

### 2.1. Participants and Procedure

A cross-sectional survey was distributed between November and early December 2020 to undergraduate students (N = 758, *M age* = 18.38±1.31; 70.1% female; see Table 1 for further demographic information) at a medium-sized Mid-Atlantic university, as well as some additional undergraduate students from other universities using a listserv. The survey included questions about dietary intake, eating behaviors, social support, overall psychological health status, and COVID-19-related stress during the Fall mid-semester. Participants were entered into a draw to win a $50 Amazon gift card upon completing the survey. The study was approved by the Johns Hopkins Institutional Review Board (IRB: NA_00092328).

### 2.2. Measures

#### 2.2.1. Demographics and Self-Reported Weight and Height

Standard demographic information, including age, race, sex assigned at birth, and year in university, was collected. Participants were also asked to report their weight and height, which were used to calculate body mass index (BMI).

#### 2.2.2. COVID-19-Related Stress

To measure COVID-19-related stress, questions were adapted from a previous study (Sadler et al., 2021), focusing on three main components:

- **Academic Stress Induced by the COVID-19 Pandemic (ASIP):** Stress related to factors such as compulsory online learning, the negative impact of isolation measures on academic performance, and changes in career prospects.
- **Financial Stress Induced by the COVID-19 Pandemic (FSIP):** Stress related to factors such as job loss, financial instability, and health insurance costs.
- **COVID-19 Exposure-Related Stress (CERS):** Stress related to concerns about contracting COVID-19 personally or someone they know being affected.

The questions used to measure each stressor category are presented in Table 2. The final measure consisted of 19 items, grouped as follows:

- **ASIP Score:** 10 items, Cronbach’s alpha = 0.88;
- **FSIP Score:** 6 items, Cronbach’s alpha = 0.78;
- **CERS Score:** 3 items, Cronbach’s alpha = 0.80.

The mean score for each stress variable was used in the analyses.

#### 2.2.3. Dietary Intake

Students reported their consumption of various food categories in the past 7 days as described in Sadler et al. (2021). Participants estimated their frequency of consuming sweets/desserts (e.g., chocolate, cookies, doughnuts, ice cream), chips/savory snacks (e.g., regular or low-fat chips, salty snacks), fast food (e.g., McDonald’s), and servings of fruits and vegetables. Response ranged from “Never” to “6 or more times per day.” Intake was reported as weekly frequency or servings. A “junk food intake” variable was calculated by summing servings of sweets, savory snacks, fast foods, and sweetened beverages consumed.

#### 2.2.4. Eating Behaviors

Students reported perceived changes since before the semester in the frequency of eating, the amount of food eaten, and health of diet. They also reported on late-night eating behavior in the past 7 days. Four subscales from the Adult Eating Behavior Questionnaire (AEBQ) (Hunot et al., 2016) assessed food responsiveness, satiety responsiveness, emotional overeating, and emotional undereating, using a 5-point Likert scale (1 = Strongly Disagree to 5 = Strongly Agree). An adapted Revised Emotional Eating Scale with Boredom (REES-B) (Koball et al., 2012) assessed emotional eating related to four emotions (“bored,” “sad,” “anxious,” and “stressed”) on a 5-point scale from “Never” to “Very Often,” with average scores calculated.

#### 2.2.5. Psychological Health

Stress levels were measured using the 4-item Perceived Stress Scale (PSS-4) (Herrero & Meneses, 2006), which asked about perceived control, confidence, and feelings of overwhelm. Responses ranged from “Never” to “Very Often,” and average scores were calculated. Depression was assessed with the 10-item Center of Epidemiologic Studies Depression Scale (CES-D-10) (Andresen et al., 1994), where participants indicated the frequency of depressive symptoms over the past week.

#### 2.2.6. Social Support

The Perceived Support Questionnaire (PSQ) (Lin et al., 2019) assessed perceptions of social security, safety, and a personal community of support. Participants rated six statements on a 5-point scale (1 = Strongly Disagree to 5 = Strongly Agree).

A more detailed overview of all measures and questionnaire is provided in the Supplementary Materials (link to Supplementary Data).

### 2.3. Statistical Analyses

Statistical analyses were conducted using IBM SPSS Statistics version 27 (IBM SPSS Statistics for Windows). Due to the nonparametric distribution of most responses, Spearman’s correlations were used to assess bivariate relationships between COVID-19-related stress and dietary intake and eating behaviors. ASIP, FSIP, and CERS variables were categorized into Low, Moderate, and High Stress based on tertiles. Chi-square tests (χ^2^) examined associations between stress levels and perceived changes in eating behaviors from pre- to mid-semester. Additional chi-square tests assessed sex-based differences in perceived changes in eating behaviors.

## 3. Results

### 3.1. Sample Characteristics

Sample characteristics are presented in Table 1. The distribution of participants across university years was relatively even, with slightly more freshmen (34.6%). The sample was predominantly female (70.1%) and consisted largely of first-year undergraduate students (34.6%), with a mean age of 18.38±1.31 years. The majority of participants fell within the healthy weight range (68.8%), with a mean BMI of 22.28±3.11.

Psychological health and social support measures and related statistics are reported in Table 3. The highest mean scores on pandemic-related stress were observed for ASIP (3.78±0.85), followed by CERS (3.46±1.02) and then FSIP (2.12±0.89).

### 3.2. Relationship between COVID-19-Related Stress and Dietary Intake

Correlations between COVID-19-related stress and dietary intake in the past 7 days are described below (Table 4).

#### 3.2.1. ASIP

As shown in Table 4, higher ASIP was correlated with increased intake of junk food (ρ = 0.092, p < 0.05), fast food (ρ = 0.13, p < 0.05), and sweets (ρ = 0.091, p < 0.05) in the past 7 days. Among females, higher ASIP was correlated with increased consumption of fast food (ρ = 0.19, p < 0.001) and sweets (ρ = 0.14, p < 0.05) and decreased consumption of fruits and vegetables (ρ = −0.12, p < 0.05), while no significant associations were observed for males.

#### 3.2.2. FSIP

FSIP was positively associated with fast food intake (ρ = 0.10, p < 0.05) in the past 7 days (Table 4). Among females, higher FSIP correlated with increased fast food consumption (ρ = 0.14, p < 0.05) and decreased fruit and vegetable intake (ρ = −0.11, p < 0.05). No significant associations were observed for males.

#### 3.2.3. CERS

CERS showed no significant overall associations with dietary intake in the past 7 days for any food group (Table 4). However, one significant relationship was observed among males, such that higher CERS was correlated with lower sweets intake (ρ = −0.15, p < 0.05).

### 3.3. Relationship between COVID-19-Related Stress and Eating Behaviors

Correlations between COVID-19-related stress and eating behaviors, including emotional eating, emotional overeating, food responsiveness, and late-night eating, are described below (Table 5a and Table 5b).

#### 3.3.1. ASIP

Higher ASIP was correlated with heightened scores on scales assessing maladaptive eating behaviors. Specifically, ASIP was positively associated with emotional overeating (ρ = 0.16, p < 0.001) and with eating in response to a range of emotions, such as boredom, worry, nervousness, and stress (ρ = 0.34, p < 0.001). A positive correlation between ASIP and food responsiveness (ρ = 0.24, p < 0.05) was observed among males only, while a positive correlation with emotional overeating was found among females only (ρ = 0.16, p < 0.05). Additionally, higher ASIP was significantly associated with an increased frequency of late-night eating episodes, as indicated by greater food consumption after dinner (β = 26.79, p < 0.001).

#### 3.3.2. FSIP

Higher FSIP was correlated with eating in response to a range of emotions (ρ = 0.26, p < 0.001), emotional overeating (ρ = 0.16, p < 0.001), and food responsiveness (ρ = 0.11, p < 0.05). The correlations for eating in response to emotions and emotional overeating were apparent in both males and females, whereas the association with food responsiveness was observed only in males (ρ = 0.22, p < 0.05) and not in females. Similar to ASIP, higher FSIP was associated with an increased occurrence of late-night eating episodes, as reflected by elevated food intake after dinner (β = 25.57, p < 0.001).

#### 3.3.3. CERS

Higher CERS was correlated with eating in response to a range of emotions (ρ = 0.17, p < 0.001), emotional overeating (ρ = 0.086, p < 0.05), and food responsiveness (ρ = 0.12, p < 0.05) (Table 4). A positive association between CERS and food responsiveness was observed in males (ρ = 0.17, p < 0.05) but not in females.

### 3.4. Perceived Changes in Eating Behaviors

Correlations between COVID-19-related stress and perceived change in eating behaviors from pre-semester to mid-semester, including changes in diet quality, frequency of eating, and the amount of food consumed are described below (Table 6). Higher ASIP was associated with a perceived less healthy diet (β = 15.94, p = 0.003) since before the semester started. Furthermore, higher ASIP was associated with changes in the frequency of eating (β = 26.79, p < 0.001), with students experiencing greater academic stress reporting both increases and decreases in their eating frequency. Higher FSIP was associated with a perceived lower amount of food consumed (β = 10.43, p = 0.034), and a perceived lower eating frequency (β = 15.45, p = 0.004). No associations of CERS with perceived change from pre- to mid-semester were observed.

Further analyses examining sex differences (Table 7) revealed notable variations in eating behavior changes during the COVID-19 period. Females were more likely to report changes in both the amount of food consumed (β = 8.55, p = 0.014) and the frequency of eating (β = 6.88, p = 0.032), whether increases or decreases. In contrast, males were more likely to report stability in their eating behaviors over the same period.

## 4. Discussion

We aimed to investigate the impact of COVID-19-related stress on the diet and eating behaviors of US college students during the Fall 2020 semester and explore sex differences within the sample. Our primary research hypothesis was that COVID-19-related stress levels would be associated not only with unhealthy dietary patterns but also with potentially maladaptive eating behaviors.

Consistent with our prediction, our primary findings indicate that several different types of COVID-19-related stress was associated with potentially maladaptive eating behaviors and less healthy dietary profiles. Specifically, COVID-19-related academic and financial stress were positively associated with fast food consumption and negatively associated with fruit and vegetable intake in females. All three COVID-19 stressors — academic (ASIP), financial (FSIP), and COVID-19 exposure (CERS) — were positively associated with emotional eating in both males and females. Additionally, stress was positively associated with food responsiveness in males.

ASIP emerged as the most influential COVID-19-related stressor (Mean ASIP = 3.78±0.85, range: 1–5). ASIP components included changes in class formats, difficulty concentrating on schoolwork and activities, social isolation, and concerns about the impact on future careers. In line with our findings, another study on 843 US college students reported a link between academic stress levels and worsened psychological health as a consequence of COVID-19 (Barbayannis et al., 2022). A likely cause of this is the abrupt transition from traditional in-person classroom settings to remote learning, which led to isolation, a decrease in outdoor extracurricular activities and interpersonal exchange, and disturbances in sleep patterns, likely partly driven by (and a contributor to) the concurrent increases in stress, anxiety, and depression among college and high education students (Nano et al., 2022; Son et al., 2020; Tahir et al., 2021).

We Specifically found that COVID-19-related academic stress was positively associated with junk food consumption and negatively associated with fruit and vegetable intake, particularly among females. Although our findings indicate a poorer dietary profile under pandemic-related stress, studies in other populations have reported the opposite, potentially reflecting pre-existing differences in nutrition and daily dietary patterns. For instance, one study found increased unhealthy food consumption among Italian students, while junk food consumption decreased among French students (Caso et al., 2020). Similarly, studies in Spain and Croatia observed increased vegetable and fruit consumption during the lockdown, with students adhering more closely to Mediterranean Diet guidelines than in pre-pandemic times (Dragun et al., 2020; Imaz-Aramburu et al., 2021). These contradictory findings highlight the influence of cultural context (e.g., dietary patterns) and cross-country differences in pandemic responses. Meanwhile, we found that increased ASIP was linked to greater emotional eating in both males and females. This aligns with pre-pandemic reports showing that low academic self-esteem and increased academic worries are more prevalent among emotional eaters than non-emotional eaters (Chamberlin et al., 2018).

Stress related to the potential consequential risks of virus exposure (i.e. CERS), such as concerns about one’s own or loved ones’ health after having contracted COVID-19, was rated high in our study (Mean CERS = 3.46±1.02, range: 1–5). Exposure stress was also positively associated with emotional eating and food responsiveness, especially in males, indicating a degree of psychological deterioration. However, the associations with emotional eating were weaker than those observed with academic stress. Nevertheless, our results align with other findings among emerging adults in the US, which reported weaker associations between pandemic-related health stress (both self and for close others) and impaired psychological health compared to financial and educational-related stress in this population (Kujawa et al., 2020).

Financial stress induced by the pandemic (FSIP) scored the lowest, as compared to other COVID-19-related stressors, among college students (Mean FSIP = 2.12±0.89, range: 1–5). This is likely because many college students in our sample were relying primarily on household income, student loans, and scholarships and were not (yet) working as full-time employees, buffering them from the immediate impacts of financial stress. However, despite this relatively lower financial stress, significant associations with dietary intake were observed. Specifically, FSIP was negatively associated with fruit and vegetable intake and positively associated with fast food intake among females, highlighting a shift toward less healthy and more affordable food options. These effects are likely influenced by broader systemic factors, including the economic challenges, food access disparities, and inflation brought about by COVID-19. Notably, during the pandemic, grocery prices for fresh fruits and vegetables rose disproportionately compared to the prices of fast food in the US, potentially intensifying these dietary shifts (Volpe et al., 2024). Such economic disparities were particularly evident in low-income school districts, which faced significant challenges in accessing primary goods and emergency nutrition programs during the pandemic, contributing to increased malnutrition rates and poorer dietary variety among students (Hall et al., 2022; McLoughlin et al., 2020). Additionally, lower family income was found to be associated with greater emotional eating in a population of healthy Saudi Arabian female students, suggesting that the financial environment can indeed increase the risk of potentially pathological eating behaviors (Al-Musharaf, 2020). In our study, higher levels of FSIP were linked to a perceived reduction in food consumption and less frequent eating, further indicating food insecurity among university students experiencing greater stress over financial situations (Bruening et al., 2018).

Our sample consisted predominantly of healthy-weight college students (72%). This is noteworthy since a large amount of the existing literature linking emotional eating and stress suggests that employing food to manage distress is more common among individuals with obesity and overweight (Burnatowska et al., 2022; Cecchetto et al., 2021; Dakanalis et al., 2023). The effects we observed may therefore potentially be stronger in a sample including a greater proportion of individuals with higher body weight.

In our investigation, we additionally explored sex differences in the impacts of pandemic-related stress on diet and eating behaviors. Our results suggest female students appeared to be slightly more susceptible to expressing distress associated with the pandemic via maladaptive eating habits and unhealthy diets compared to males. This predominant female vulnerability is coherent with previous analogous evidence within young populations (Ulloa et al., 2022).

This study has several limitations. First, being cross-sectional, it is unable to determine the causal relationship of interest. Second, there is potential for recall bias, as some questions required participants to remember their behaviors prior to the semester in order to compare them with the current behaviors (mid-semester). Third, our study largely relied on responses from an private Mid-Atlantic university where ethnic diversity is high but the socioeconomic status of participants is generally higher than the average American college student (Gregor Aisch et al., 2017; JHU Report on Undergradute Student Composition, 2023). The higher socioeconomic status of these participants may explain why financial COVID stress ranked as the lowest concern. Possibly, in a different population, financial stress could have been a more prominent source of COVID-19-related stress. Also, these findings might reflect a US-specific situation, considering that US, and even each state within the US, had a different approach to regulations compared to Europe. Lastly, our study did not investigate whether the relationship between COVID-19-related stressors and eating behaviors differs by weight status.

In conclusion, our findings suggest that the unique concerns introduced by the abrupt and stressful COVID-19 pandemic, and in particular academic stress, significantly impacted not only dietary quality but also eating behaviors (especially eating in response to emotions and late-night eating) of college students, with effects varying between sexes. These results illuminate young adults’ susceptibility to different types of stress generated by a population-level health event and the detrimental impact of such stress on dietary health, as well as the development of potentially pathological eating behaviors. These behaviors may carry long-term risks, including an increased likelihood of metabolic syndrome and depression (Gallant et al., 2012; Graybeal et al., 2023; Gu et al., 2020; Tuncay & Sarman, 2024). Our results suggest that support measures aimed at tackling stress and providing resources for stress management and maintenance of health behaviors during stress are warranted. Such interventions could be of value not only in extreme circumstances but to help students navigate normative academic and educational environments while maintaining physical and psychological health.

## Figures and Tables

**Table 1:** Sample characteristics.

		N (%)
Year in university (n = 758)	Freshman/First-year undergraduate	262 (34.6%)
	Sophomore/Second-year undergraduate	171 (22.6%)
	Junior/Third-year undergraduate	175 (23.1%)
	Senior/Fourth-year undergraduate	143 (18.9%)
	Fifth year or more undergraduate	7 (0.9%)
Sex assigned at birth (n = 757)	Male	224 (29.6%)
	Female	531 (70.1%)
	Prefer not to say	2 (0.3%)
Race (n=757)	Black	68 (9.0%)
	White	209 (27.6%)
	Hispanic/Latin (identify solely this way)	41 (5.4%)
	Native Hawaiian/ Pacific Islander	1 (0.1%)
	Asian	293 (38.7%)
	Mixed	125 (16.5%)
	Other	8 (1.1%)
	Decline to Answer	12 (1.6%)
Age group (n = 757)	17	15 (2.0%)
	18	223 (29.5%)
	19	173 (22.9%)
	20	180 (23.8%)
	21	134 (17.7%)
	22	28 (3.7%)
	23	3 (0.4%)
	Older than 25	1 (0.1%)
BMI (excluding outliers, n = 481)	Underweight, BMI < 18.5	40 (8.3%)
	Healthy weight, BMI 18.5–24.9	331 (68.8%)
	Overweight, BMI 25.0–29.9	99 (20.6%)
	Obesity, BMI >30	11 (12.3%)

Note: Totals for each characteristic may not equal the overall sample size of 758 due to missing or incomplete data for some variables. BMI: body mass index.

**Table 2:** COVID-19-Related Stressors Measures a) ASIP, b) FSIP, and c) CERS.

a) ASIP question: How stressed are you about the following?
1. My academic performance will be or is being negatively impacted by the COVID-19 pandemic
2. The new layout of my classes (all online, all in-person, online & in-person) will affect or has affected my ability to succeed in courses
3. Being unable to concentrate on school activities due to your learning environment OR fearing that you will be unable to concentrate on school activities in the future
4. Being unable to complete assignments due to your learning environment OR fearing you will be unable to complete assignments due to your learning environment
5. Being unable to get a job/internship
6. My GPA will drop due to the circumstances of this semester (COVID-19 pandemic, financial stability, bad learning environment, inability to work at home, etc.)
7. Decreased productivity with schoolwork or school-related activities
8. Ongoing need for social isolation due to COVID-19 pandemic
9. Future job/career prospects
10. Changing plans as to reopening of schools (i.e., uncertainty of knowing whether the school would have in-person, online, or hybrid(in-person/online) classes)
b) **FSIP question: How stressed are you about the following?**
1. Not being able to pay for basic needs (rent/mortgage, food, etc.)
2. Losing my job
3. Someone I depend upon for income losing their job
4. I will be unable to access medical care for myself or my family
5. My family/I will be unable to pay for my college tuition
c) **CERS question: How stressed are you about the following?**
1. I will get COVID-19
2. A relative (e.g. grandparent) or close family friend will get COVID-19
3. Someone I live with will get COVID-19

Note: ASIP: academic stress induced by the COVID-19 pandemic; FSIP: financial stress induced by the COVID-19 pandemic; and CERS: COVID-19 exposure-related stress. Response options for all questions were rated on a 5-point Likert scale: Not at all (1), Slightly (2), Somewhat (3), Moderately (4), and Extremely (5).

**Table 3:** Median and Mean (SD) Scores for Psychological Health and Social Support Variables.

Variables		Median	Mean (SD)
Pandemic-related stressors	ASIP	4.00	3.78 (0.85)
	FSIP	2.00	2.12 (0.89)
	CERS	3.67	3.46 (1.02)
Psychological health	CES-D-10	14.00	14.50 (6.10)
	PSS-4	3.25	3.30 (0.73)
Social support	PSQ	22.00	22.20 (4.60)

Note: SD: standard deviation; CERS: COVID-19 exposure-related stress (range: 1–5); FSIP: financial stress induced by the COVID-19 pandemic (range: 1–5); ASIP: academic stress induced by the COVID-19 pandemic (range: 1–5); CES-D-10: Center of Epidemiologic Studies Depression Scale (range: 10–40); PSS-4:4-item Perceived Stress Scale (range: 1–5); PSQ: Perceived Support Questionnaire (range: 6–30).

**Table 4:** Spearman’s Correlations (ρ) and 95% CIs Between COVID-19-Related Stressors and Dietary Intake Over the Past 7 Days in Whole Sample,Males, and Females.

COVID-19-Related Stressors	Junk Food Intake			Fruit and Vegetable Intake			Fast Food Intake		Sweets Intake			
	Whole sample	Males	Females	Whole sample	Males	Females	Whole sample	Males	Females	Whole sample	Males	Females
**ASIP**	0.092* **(.01, .17)**	−0.026 (−.18, .13)	0.16* **(.06, .26)**	−0.037 (−.12, .05)	0.11 (−.05, .26)	−0.12* **(−.22, −.02)**	0.13* **(.04, .21)**	0.014 (−.14, .17)	0.19** **(.09, .28)**	0.091* **(.0.17)**	−0.007 (−.16, .14)	0.14* **(.04, .23)**
**CERS**	0.001 (−.08, .08)	−0.126 (−.27, .03)	0.062 (−.04, .16)	−0.011 (−.10, .07)	0.053 (−.10, .21)	−0.055 (−.15, .05)	0.048 (−.04, .13)	0.063 (−.09, .22)	0.055 (−.05, .16)	−0.003 (−.09, .08)	−0.15* **(−.30, .00)**	0.058 (−.04, .16)
**FSIP**	0.016 (−.07, .10)	−0.018 (−.17, .13)	0.047 (−.05, .15)	−0.037 (−.12, .05)	0.063 (−.09, .22)	−0.11* **(−.21, −.01)**	0.10* **(.02, .19)**	0.038 (−.12, .19)	0.14* **(.04, .24)**	0.035 (−.05, .12)	−0.015 (−.17, .14)	0.060 (−.04, .16)

Note: ASIP: academic stress induced by the COVID-19 pandemic; FSIP: financial stress induced by the COVID-19 pandemic; CERS: COVID-19 exposure-related stress.

Significant differences (p < 0.05) are bolded.

* p < 0.05,

** p < 0.001.

Junk food includes sweet and savory snacks, fast food, and sugar-sweetened beverages.

Confidence intervals (CI) are presented in parentheses.

**Table 5:** a. Spearman's Correlations (ρ) and 95% CIs Between COVID-19-Related Stressors and Eating Behaviors in Whole Sample, Males, and Females.

COVID-19-Related Stressors	REES B Emotional Eating			AEBQ Emotional Overeating			AEBQ Food Responsiveness		
	Whole sample	Males	Females	Whole sample	Males	Females	Whole sample	Males	Females
**ASIP**	0.34** **(.25, .41)**	0.28** **(.13, .42)**	0.36** **(.26, .44)**	0.16** **(.07, .24)**	0.13 (−.03, .28)	0.16* **(.06, .26)**	0.078 (−.01, .16)	0.24* **(.09, .39)**	0.003 (−.10, .11)
**CERS**	0.17** **(.08, .25)**	0.17* **(.01, .32)**	0.16* **(.06, .26)**	0.086* **(.00, .17)**	0.066 (−.09, .22)	0.093 (−.01, .19)	0.12* **(.03, .20)**	0.17* **(.01, .31)**	0.088 (−.01, .19)
**FSIP**	0.26** **(.18, .34)**	0.28** **(.12, .42)**	0.25** **(.15, .34)**	0.16** **(.07, .24)**	0.19* **(.03, .34)**	0.14* **(.04, .24)**	0.11* **(.02, .19)**	0.22* **(.06, .36)**	0.061 (−.04, .16)

Note: ASIP: academic stress induced by the COVID-19 pandemic; FSIP: financial stress induced by the COVID-19 pandemic; CERS: COVID-19 exposure-related stress; REES B: Revised Emotional Eating Scale with Boredom and Emotions Experienced; AEBQ: Adult Eating Behavior Questionnaire.

Significant differences (p < 0.05) are bolded.

* p < 0.05,

** p < 0.001.

Confidence intervals (CI) are presented in parentheses.

**Table 5:** b. Chi-Square Tests (χ^2^) of Associations Between COVID-19-Related Stressors (tertiles) and Late-Night Eating in the Past 7 Days.

ASIP and Late-Night Eating				
*ASIP*	*Less than half of diet consumed after dinner*	*About half of diet consumed after dinner*	*More than half of diet consumed after dinner*	χ^2^ = 26.79, p< 0.001
Low stress	172 (39.8%)	25 (27.2%)	9 (16.1%)	
Moderate stress	150 (34.7%)	37 (40.2%)	16 (28.6%)	
High stress	110 (25.5%)	30 (32.6%)	31 (55.4%)	
FSIP and Late-Night Eating				
*FSIP*	*Less than half of diet consumed after dinner*	*About half of diet consumed after dinner*	*More than half of diet consumed after dinner*	χ^2^ = 25.57, p< 0.001
Low stress	165 (38.2%)	26 (28.3%)	14 (25.0%)	
Moderate stress	145 (33.6%)	24 (26.1%)	10 (17.9%)	
High stress	122 (28.2%)	42 (45.7%)	32 (57.1%)	

Note: ASIP: academic stress induced by the COVID-19 pandemic; FSIP: financial stress induced by the COVID-19 pandemic.

**Table 6:** Chi-Square Tests (χ^2^) of Associations Between COVID-19-Related Stressors (tertiles) and Perceived Change in Eating Behaviors from Pre- to Mid-Semester.

ASIP and Perceived Change in Health of Diet				
*ASIP*	*Eating less healthily*	*Eating equally healthy*	*Eating more healthily*	χ^2^ = 15.94, p = 0.003
Low stress	81 (28.8%)	63 (45.7%)	51 (42.5%)	
Moderate stress	100 (35.6%)	43 (31.2%)	40 (33.3%)	
High stress	100 (35.6%)	32 (23.2%)	29 (24.2%)	
ASIP and Perceived Change in Frequency of Eating				
*ASIP*	*Eating less frequently*	*Same eating frequency*	*Eating more frequently*	χ^2^ = 26.79, p<0.001
Low stress	84 (32.1%)	52 (50.0%)	58 (33.7%)	
Moderate stress	91 (34.7%)	33 (31.7%)	59 (34.3%)	
High stress	87 (33.2%)	19 (18.3%)	55 (32.0%)	
FSIP and Perceived Change in Amount of Food Consumed				
*FSIP*	*Eating less food*	*Eating about the same amount of food*	*Eating more food*	χ^2^ = 10.43, p = 0.034
Low stress	64 (28.8%)	62 (41.1%)	62 (37.6%)	
Moderate stress	66 (29.7%)	46 (30.5%)	54 (32.7%)	
High stress	92 (41.4%)	43 (28.5%)	49 (29.7%)	
FSIP and Perceived Change in Frequency of Eating				
*FSIP*	*Eating less frequently*	*Same eating frequency*	*Eating more frequently*	χ^2^ = 15.45, p = 0.004
Low stress	77 (29.4%)	51 (49.0%)	60 (34.9%)	
Moderate stress	83 (31.7%)	22 (21.2%)	60 (34.9%)	
High stress	102 (38.9%)	31 (29.8%)	52 (30.2%)	

Note: ASIP: academic stress induced by the COVID-19 pandemic; FSIP: financial stress induced by the COVID-19 pandemic.

**Table 7:** Chi-Square Tests (χ^2^)D for Associations Between Sex and Perceived Change in Eating Behaviors from Pre- to Mid-Semester.

Relationship between Sex and Perceived Change in Amount of Food Consumed				
	*Eating Less Food*	*Eating About the Same Amount of Food*	*Eating More Food*	χ^2^ = 8.55, p = 0.014
Male	52 (23.4%)	56 (37.1%)	53 (32.1%)	
Female	170 (76.6%)	95 (62.9%)	112 (67.9%)	
Relationship between Sex and Perceived Change in Frequency of Eating				
	*Eating Less Frequently*	*Same eating frequency*	*Eating more frequently*	χ^2^ = 6.88, p = 0.032
Male	70 (26.7%)	42 (40.4%)	49 (28.5%)	
Female	192 (73.3%)	62 (59.6%)	123 (71.5%)	

## Data Availability

The datasets used and/or analysed during the current study are available from the corresponding author on reasonable request.

## Abbreviations

BMI: Body Mass Index
ASIP: Academic Stress Induced by the COVID-19 Pandemic
FSIP: Financial Stress Induced by the COVID-19 Pandemic
CERS: COVID-19 Exposure-Related Stress
AEBQ: Adult Eating Behavior Questionnaire
REES-B: Revised Emotional Eating Scale with Boredom
PSS-4: 4-item Perceived Stress Scale
CES-D-10: 10-item Center of Epidemiologic Studies Depression Scale
PSQ: Perceived Support Questionnaire
